# Adherence influencing factors – a systematic review of systematic reviews

**DOI:** 10.1186/2049-3258-72-37

**Published:** 2014-10-27

**Authors:** Tim Mathes, Thomas Jaschinski, Dawid Pieper

**Affiliations:** Institute for Research in Operative Medicine, Faculty of Health - School of Medicine, Witten/Herdecke University, Ostmerheimer Str. 200, Building 38, D- 51109 Cologne, Germany

**Keywords:** Adherence, Compliance, Systematic review, Oral medications, Oral therapy

## Abstract

**Background:**

Non-adherence is widespread problem. Adherence is a crucial point for the success and the safe use of therapies. The objective of this overview (review of reviews) was to identify factors that influence adherence in chronic physical conditions.

**Methods:**

A systematic literature search was performed in Medline and Embase (1990 to July 2013). Publications were screened according to predefined inclusion criteria. The study quality was assessed using AMSTAR. Both process steps were carried out independently by two reviewers. Relevant data on study characteristics and results were extracted in piloted standardized tables by one reviewer and checked by a second. Data were synthesized using a standardized quantitative approach by two reviewers.

**Results:**

Seven systematic reviews were included. Higher education and employment seem to have a positive effect on adherence. Ethnic minorities seem to be less adherent. Co-payments and higher medication cost seems to have negative effect on adherence. In contrast financial status/income and marital status seem to have no influence on adherence. The effect of therapy related factors was mostly unclear or had no effect. Only the number of different medications in heart failure patients showed the tendency of an effect. Indicators of regime complexity showed consistently a negative effect direction. Duration of disease seems to have no effect on adherence. There is the tendency that higher or middle age is associated with higher adherence. But in more than half of the reviews the effect was unclear. There is no clear effect of physical as well as mental comorbidity. Only one review showed the tendency of an effect for mental comorbidity. Also for gender the effect is not clear because the effect direction was heterogenic between and within the systematic reviews.

**Conclusion:**

The presented overview shows factors than can potentially have influence on adherence. Only for a few factors the influence on adherence was consistent. Most factors showed heterogeneous results regarding statistical significance and/or effect direction. However, belonging to an ethnic minority, unemployment and cost for the patient for their medications showed consistently a negative effect on adherence which indicates that there is a social gradient.

**Electronic supplementary material:**

The online version of this article (doi:10.1186/2049-3258-72-37) contains supplementary material, which is available to authorized users.

## Background

Adherence, can be defined as “the extent to which a patient acts in accordance with the prescribed interval and dose of a dosing regimen” [1]. Non-adherence is widespread problem in chronic conditions [2, 3]. Adherence is not only a crucial point for the success, but also for the safe and effective use of many therapies [4–6]. Moreover non-adherence can cause substantial costs [3]. In chronic conditions adherence is especially important because medication has to be taken for a long time.

Adherence is a multifactorial problem that can be influenced by various factors. The factors can be roughly divided in the following five dimensions: Social and economic, health care system, health condition, therapy and patient [3]. Furthermore non-adherence can be intentional (e.g. decided not to take because of adverse events) and non-intentional (e.g. forgetfulness).

The objective was to identify factors that influence adherence to oral medications in patients suffering from physical chronic conditions. Because of the large amount of literature in this field it is difficult for researchers and clinicians to know all relevant studies for all physical chronic conditions. Thus, we decided to perform a review of reviews (overview) which is a new form of evidence synthesis to answer the research question. An overview can provide a comprehensive picture of the evidence while keeping the information flood manageable [7]. Furthermore we decided to focus on factors that are not associated with the indication or therapy to enhance universal applicability of the results.

## Methods

### Sources

A systematic literature search was performed in MEDLINE (via Pubmed) and Embase (via Embase). The search strategy combined various terms and medical subject headings related to adherence, and oral medical medication (the full search strategies for each database are available in Additional file 1). The search was limited to a publication date after 1990. The search was performed on July the 15th 2013.

### Study selection

To be eligible for this review the studies had to meet the following inclusion criteria:

1. Patients: Adult patients with physical chronic condition (i.e. studies that analysed only children, patients with acute conditions or mental illness were excluded).
2. Medication: Oral intake.
3. Exposure: Potentially adherence influencing factor (exposure is not controlled by the investigator).
4. Outcome Adherence (right dose, right timing and/or right frequency of intake).
5. Study type: Systematic review (definition: systematic literature search in at least one electronic database and assessment of risk of bias of included studies) of quantitative studies.
6. Publication language: English or German.

It was decided to focus on factors unrelated to the health care system (e.g. type of insurances), condition (e.g. symptoms) and medication (e.g. side effects) i.e. factors that are strongly related to health care system, condition or medications were excluded. For example side effects or symptoms are strongly associated with the respective medication disease respectively and can consequently have different influence on adherence. The purpose of focusing on disease and medication unrelated factors was on the one hand to insure comparability, consistency and clarity of results and on the other hand to identify factors that are widely applicable in clinical practice. A preliminary unsystematic search was performed to identify articles on adherence influencing factors. Based on identified articles a list of potential adherence influencing factors was prepared and chosen which factors are unrelated to the disease and therefore should be included in the analysis. The decision to include a factor was made independently by two reviewers and discussed until consensus in case of discrepancies. The following factors were eligible: age, gender, ethnic status, education, employment, financial status/income, marital status/not living alone, social support, measure of intake complexity (e.g. number of tablets, number of medications, frequency of intake), duration of therapy, duration of disease, comorbidity, co-payments, medication costs, insurance status (insured/not ensured).

Furthermore it was decided to include systematic reviews only if the risk of bias of included primary studies was assessed, documented and relatable to the respective factor because an examination of the evidence for an effect of a potential adherence influencing factor is only possible considering the validity of evidence of the primary studies.

Two reviewers performed the study selection independently according to the inclusion criteria above. Titles and abstracts of all articles were screened. The full-texts of all potentially relevant articles were than obtained and screened in detail. Any differences between the reviewers were discussed until consensus. In addition to identify grey literature the reference lists of all full-texts were cross-checked and an additional search in Google scholar performed.

### Methodological quality assessment

The methodological quality of included studies was assessed using the AMSTAR instrument that was found to be valid and reliable to assess SRs [8, 9]. Each assessment question was rated with “yes”, “no”, “unclear” or “not applicable”. The instrument was applied independently by two reviewers. Disagreements were resolved in a discussion. The question “was the scientific quality of the included studies assessed and documented” was skipped because we formulated quality assessment of included studies as inclusion criterion (see inclusion criterion 5).

### Data extraction and synthesis

Data were extracted by one reviewer in standardized summary tables and were verified by a second reviewer. Any disagreements were discussed till consensus. For each systematic review, characteristics were extracted on the condition/medication (marked bold), inclusion and exclusion criteria for primary studies (only other than our applied inclusion criteria) and search period limits. Results were extracted according to the type of evidence synthesis. In the case of a narrative synthesis, results were abstracted by modified vote counting [10]. This contained data to the effect direction, all comparisons showing this effect direction, statistically significant comparisons showing this effect direction and total number of comparisons for this factor. This method has been suggested for presenting results of qualitative synthesis, overcoming problems arising when simple vote counting is used by relying either on the number of comparisons with a positive direction of effect or the number of comparisons reaching statistical significance. For all meta-analyses, pooled effect sizes and measures with confidence intervals and test and measure for statistical heterogeneity were extracted. All data in the tables were commutated so that the influence on adherence refers throughout to an increase of the respective factor independently whether the factor is positive (e.g. educational level) or negative (adverse events). A p-level of less than 0.05 was considered statistical significant. Overlaps (multiple included primary studies) were assessed in case of multiple systematic reviews on the same indication.

For all factors a summary evaluation of the influence was made. Two reviewers rated the evidence for an effect, considering the number of included systematic reviews for a comparison, the effect direction, the significance and consistency (within and between systematic reviews) of results, the methodological quality of systematic reviews and the methodological quality/risk of bias of included primary studies. If only one high quality study was included in a comparison that showed a statistical significant positive effect for a certain factor, this was rated as tendency for a positive effect. Discrepant judgments were discussed until consensus.

## Results

### Description of included systematic reviews

**Figure 1:**
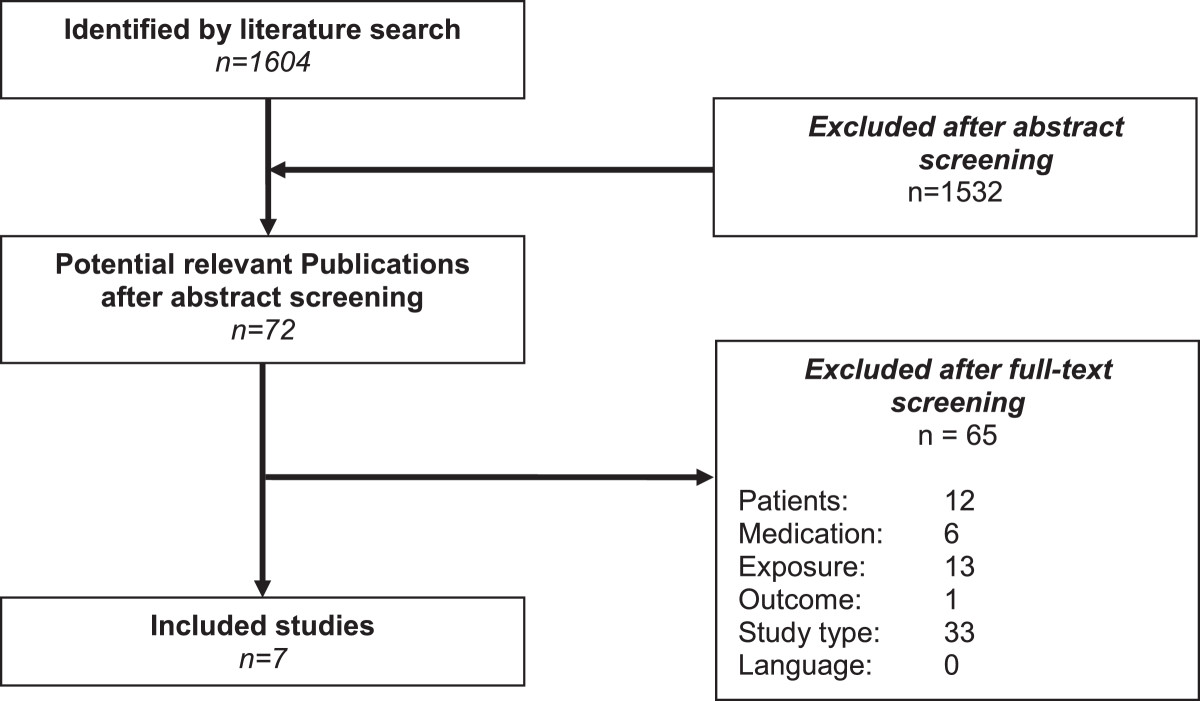
**Flow-** **chart of study selection.**

The electronic literature search resulted in 1604 hits after duplet removal (EndNote ×5). Of these 72 titles and abstracts seemed potential relevant and full-text versions of the publications were screened in detail. Seven studies met all inclusion criteria and were finally included in this overview [11–17]. The process of study selection is illustrated in the flowchart (see Figure 1). Most systematic reviews were excluded because a risk of bias assessment was not performed or the quality of evidence was not attributable to the influencing factors (inclusion criteria 5).

The included systematic reviews assessed adherence influencing factors in the following conditions/medications; chronic malignant pain, Parkinson disease, heart failure, inflammatory arthritis, prophylactic treatment of hemophilia A or B, intake of oral anti-cancer agents [11–15, 17]. Sinoott et. al did not restrict the condition or medication but included all studies that analysed public insured patients which were exposed to co-payments for medications [16]. Overlaps were not assessed, because all included systematic reviews were on different indications. The characteristics of the included systematic reviews are presented in Table 1.

**Table 1:** **Study characteristics**

Study	Search period	Inclusion criteria*
Broekmans [11]	Not limited – 12/2006	***Adult patients with chronic non*** ***-malignant pain***
		***Adult patients with prescribed pain medication***
		Original research
Daley [12]	Not limited – 01/2012	***Patients with Parkinson***
		***All ranges and duration of anti-*** ***parkinsonian treatments***
		All age ranges
		Published in English
		Presenting quantitative/qualitative data
Oosterom-Calo [13]	Not limited – 08/2010	≥***50%*** ***heart failure patients***
		Quantitative results were reported
		Studies of at least fair quality
		Evaluations of interventions were not the main purpose
		No descriptive study
		No review paper
		Published in English
Pasma [14]	Not limited – 02/2011	***Inflammatory arthritis patients***
		Used a reproducible definition or validated instrument to measure adherence
		Provided a statistical measure to reflect the strength of the association between the determinant and adherence
		No letters, editorials, reviews, RCTs, case reports, qualitative studies and opinion articles
Schrijvers [15]	Not reported – 15/2012	***Haemophilia A or B***
		***Prophylactic treatment***
		All age groups
Sinnott [16]	1946 – 09/2012	***Participants received healthcare from a public insurance scheme***
		Comparator group was the same population/similar population who either didn’t pay copayments or experienced no increase in copayment
		The intervention was copayment; either an increase in an existing copayment or the introduction of a copayment (no other types of cost-sharing, for example co-insurance)
		Studies included were randomised controlled trials, controlled before and after studies, interrupted time series designs, repeated measures designs, and cohort designs
Verbrugghe [17]	NR	***Oral anti-*** ***cancer drugs***
		Age ≥ 18
		Strong or moderate methodological quality
		Written in English, French, German or Dutch
		Original research articles published between 1990 and April 2012
		Studies not conducted in developing countries
		All study designs

*Indication & medication marked bold.

### Methodological quality of included systematic reviews

Overall the quality of included systematic reviews was moderate to high One systematic review satisfied all quality criteria [16] and two did not fulfil only one quality criterion [12, 17]. At most three items were not answered with “no” or “unclear” (three studies [11, 14, 15]). In three systematic reviews the study selection and/or data extraction was not performed in duplicate or the item was rated as unclear because of missing information [11, 14, 15]. The most frequent flaw was that a list of excluded studies was not provided. The assessment questions relating to combing findings and publication bias were rated as not applicable in 6 studies because a quantitative data-synthesis was not performed. The quality of each systematic review is presented in Table 2.

**Table 2:** **Results of the study quality assessment**

Assessment question	á priori design	Two reviewers	Literature search	Status of publication	List of studies	Study characteristics	Conclusions	Combining findings	Publication bias	Conflict of interest
Study										
Broekmans [11]	+	?	+	+	-	+	+	O	O	-
Daley [12]	+	+	+	+	-	+	+	O	O	+
Oosterom-Calo [13]	+	+	-	+	-	+	+	O	O	+
Pasma [14]	+	?	+	+	-	+	+	O	O	-
Schrijvers [15]	+	-	+	?	-	+	+	O	O	+
Sinnott [16]	+	+	+	+	+	+	+	+	+	+
Verbrugghe [17]	+	+	+	+	-	+	+	O	O	+

+ = yes; − = *no*; O = not applicable; ? = unclear.

### Effect of adherence influencing factors

The following factors were included in the analysis: age, comorbidity, co-payments, duration of disease, duration of therapy, education, ethnic status, gender, regime complexity (e.g. number of daily intake, number of tablets, frequency of intake, daytime of intake), marital status (including domestic partnerships), medication costs, different medications, social support, financial status/income, employment.

The results of individual systematic reviews are presented in Additional file 2 and the evidence synthesis of individual systematic reviews is presented in Table 3.

**Table 3:** **Evidence synthesis**

Factor	Relationship		
	Indication/therapy	Effect direction	Evidence for effect
***Social and economic***			
Education	Chronic pain	**O**	**+**
	Parkinson	↑	**O**
	Heart failure*	**O**	**O**
	Oral cancer therapy	↑	**O**
Employed	Inflammatory arthritis	**O**	**+**
Ethnic status	Parkinson	Ethnic minorities < others	**+**
	Inflammatory arthritis	White > others	**+**
	Oral cancer therapy	Non-white > others	**O**
Financial status/income	Parkinson	↑	**-**
	Heart failure*	**O**	**-** **-**
	Oral cancer therapy	↑	**O**
Married/not living alone	Parkinson	↑	**-**
	Oral cancer therapy	↕	**O**
Social support	Heart failure*	↕	**O**
	Inflammatory arthritis	↑	**O**
***Therapy related***			
Duration of therapy	Oral cancer therapy	↓	**O**
Frequency of intake	Parkinson	↓	**O**
	Heart failure*	**O**	**-** **-**
	Inflammatory arthritis	**O**	**-** **-**
Number of pills taken per day	Heart failure*	**O**	**O**
Different medications	Chronic pain	↕	**O**
	Parkinson	↓	**O**
	Inflammatory arthritis	↓	**O**
	Oral cancer therapy	↓	**O**
	Heart failure*	**O**	**+**
Taking medication at meals	Oral cancer therapy	↑	**O**
***Disease related***			
Duration of disease	Chronic pain	**O**	**-** **-**
	Inflammatory arthritis	↓	**-**
	Oral cancer therapy	↓	**O**
***Patient related***			
Age	Chronic pain	↑	**O**
	Parkinson	↑	**+**
	Heart failure*	↑	**+**
		35-56 > others	**+**
	Inflammatory arthritis	↑	**O**
		55-64 > others	**+**
	Hemophilia	↑	**O**
	Oral cancer therapy	↕	**O**
Comorbidity (not specified)	Inflammatory arthritis	↑	**O**
Comorbidity (physical)	Heart failure*	↕	**O**
Comorbidity (mental)	Parkinson	↓	**O**
	Heart failure*	↓	**+**
Gender (female)	Chronic pain	↑	**+**
	Heart failure*	↕	**O**
	Oral cancer therapy	↕	**O**
***Health care system factors***			
Co-payments	Inflammatory arthritis	↓	**+**
	Not restricted	↓	**++**
	Oral cancer therapy	↓	**O**
Medication costs	Inflammatory arthritis	↓	**+**
	Oral cancer therapy	↓	**O**

Effect direction.

↑: positive (all studies showed a positive effect on adherence); ↓: negative (all studies showed a negative effect on adherence); ↕: heterogenic (i.e. at least one study showed a contrary effect direction); O: unclear reported/ not reported.

Evidence for effect.

++: clear effect.

+: tendency of effect.

--: no effect.

-: tendency of no effect.

O: unclear effect.

*Inclusion criteria for studies: ≥50% of population are heart failure patients.

### Social and economic factors

The evidence for effect of education was mostly judged as unclear. Only in chronic malignant pain there is a tendency that education has effect on adherence [11]. However the effect direction was not reported in this systematic review. In the systematic reviews that reported effect direction, education showed a positive influence on adherence [13, 17].

Employment was only analysed in the systematic review on inflammatory arthritis [14]. There is a tendency of effect but the effect direction was not reported.

In Parkinson diseases there is the tendency that ethnic minorities are less adherent [12]. In inflammatory arthritis whites seem to be more adherent [14].

Financial status seems to have no effect on adherence. The three systematic reviews that analysed this outcome showed no effect, the tendency of no effect, or unclear effect respectively [12, 13, 17].

The effect of social support was unclear in both systematic reviews that analysed this exposure [13, 14].

### Therapy related factors

Duration of therapy was only analysed in the systematic review on oral anticancer agents [17]. The effect direction was negative. But the evidence for effect was unclear.

The effect direction of frequency of intake was negative in one primary study on patients with Parkinson diseases [12]. However, the study was not statistical significant. There is no effect of frequency of intake on adherence in patients with heart failure or inflammatory arthritis patients [14].

The effect of tablets taken per day in heart failure patients is unclear, because one of the two studies that analysed this factor was statistical significant and one was not. Moreover effect direction was not reported [13].

Most of the systematic reviews that analysed intake of different medications showed a negative effect on adherence (Parkinson, oral anticancer therapy, inflammatory arthritis) [12, 14, 17]. In patients with chronic malignant pain the effect direction was heterogenic for this factor [11]. For taking different medications there was some evidence for effect in heart failure patients. But effect direction was unclear [13].

Taking medications at meals showed a positive effect direction on adherence in patients taking oral anticancer agents. The evidence for effect was unclear [17].

Duration of diseases seems to have no influence on adherence in patients with chronic malignant pain and tendentially not in patients with inflammatory arthritis [11, 14]. For patients taking oral anticancer agents the effect direction was negative, but evidence for effect was unclear [17].

### Patient related factors

With one exception [17], all studies showed a positive effect of higher age or middle age on adherence. There was a tendency for effect in patients with Parkinson and heart failure [12, 13]. In patients with inflammatory arthritis the effect of higher age was unclear. Patients aged between 35–56 showed higher adherence than other age groups in one study [14]. For the other indications (chronic malignant pain, hemophilia, oral anticancer therapy) the evidence of effect was unclear [11, 15, 17].

The evidence for effect of comorbidity was mostly unclear [12–14]. Only in heart failure patients there was the tendency of negative effect of mental comorbidities [13].

The effect direction of gender was heterogeneous in patients with heart failure and patients on oral anticancer therapy [13, 17]. In patients with chronic malignant pain there was the tendency of higher adherence in women [11].

### Health care system factors

Co-payments and medication costs showed a negative effect direction in all systematic reviews. For both factors there was a tendency of effect in inflammatory arthritis patients and unclear effect in patients taking oral anticancer agents [14, 17]. The meta-analysis on co-payments in public insurance systems showed a clear negative effect [16].

## Discussion

Seven relevant systematic reviews could be identified that satisfied all inclusion criteria. The quality of the systematic reviews was moderate to high.

Of the social economic factors higher education and employment seem to have a positive effect on adherence. Moreover ethnic minorities seem to be less adherent. In contrast financial status/income and marital status seem to have no influence on adherence. The effect of therapy related factors was mostly unclear or had no effect. Only the number of different medications in heart failure patients showed the tendency of an effect. However indicators of regime complexity showed consistently a negative effect direction. Duration of disease seems to have no effect on adherence. There is the tendency that higher or middle age is associated with higher adherence. But in more than half of the reviews the effect was unclear. There is no clear effect of physical as well as mental comorbidity. Only one review showed the tendency of an effect for mental comorbidity. Also for gender the effect is not clear because the effect direction was heterogenic between and within the systematic reviews. Co-payments and higher medication cost seems to have negative effect on adherence.

The results of the excluded systematic reviews on other chronic conditions are broadly in accordance with the results of the presented analysis. However most of these did not assess the risk of bias of included primary studies and results could therefore not be assessed systematically [18–23].

Although the quality of systematic reviews was moderate to high, there is a loss of information in our overview because of poor reporting in the systematic reviews. Thus, we often had to rate the evidence for effect as unclear not because of a “real” lack of evidence. For example it was not possible to include the effect size and number of included patients in the evidence synthesis because with one exception [16] none of the studies provide information on these. It appears that some studies report only significant comparisons. These impression is also in accordance with prior research [24]. For such reviews that did not mention the total number of comparisons for a certain factor but only the statistical significant comparisons it is impossible to make a summary estimation of effect. Consequently the evidence for effect in the systematic review of Daley et al. and Verbrugghe et al. had to be judged as unclear, throughout [12, 17].

Another limitation is that there were no information on adjustments of analysis, although confounding is a major problem in observational study designs [25, 26]. So data had to be extracted irrespective of the adjustments with might cause some of the heterogeneity of results. Furthermore no information can be derived on the relation between the factors. Thus, assumptions like that the effect of co-payments and medication costs is stronger in low income groups cannot be proven.

In continuous variables source of heterogeneity are probably the different categorizations. For example studies that analysed age with different categorizations showed higher adherence in the middle aged. In contrast studies that analysed age as linear showed often no statistical significance results and effect directions were partly even conflicting. Also for the factors that describe the complexity of intake regime different definitions and categorizations are probably a source for heterogeneity of results (e.g. frequency of intake, number of different medications). For such factors a well-chosen categorization (e.g. by testing different categorizations), definition and clear reporting of analysis is warranted for a substantial evaluation.

In the overview symptomatic as well as asymptomatic conditions were considered. Research has shown, probably because of the lower perceived importance of taking medications correctly that adherence is lower in asymptomatic conditions than in symptomatic conditions [27, 28]. The differences in adherence might be another reason for between study heterogeneity that is not controlled for.

A further probable source for heterogeneity is that there is no differentiation between intentional and non-intentional adherence. Indeed prior research revealed that non-adherence is mostly non-intentional [29]. However, the influence of an factor can differ between intentional and non-intentional adherence [30].

The overview has some methodological limitations. Firstly there is a risk for publication bias because we did not search other languages than English and German. Secondly the overview focuses only on therapy implementation. I.e. initiation and discontinuation of therapy were not considered [31]. Thirdly we assessed the included systematic reviews of prognostic factors with AMSTAR, which was originally developed for the quality assessment of systematic reviews of health interventions and which was only validated in randomised controlled trials.

These review aimed to provide a comprehensive overview of factors that can have influence on adherence for a wide variety of indications. Surprisingly, despite the mass of literature on adherence we could identify only systematic reviews on seven different chronic physical conditions that satisfied all inclusion criteria. The results possibly can be transferred to other conditions because we considered only condition and therapy unrelated factors in physical chronic conditions. This suggestion is confirmed by the results of systematic reviews on other chronic conditions [18–23]. However, it should be considered in the interpretation of data that the generalizability is limited because of this fact.

Only condition and therapy unrelated factors were analysed in this overview. There are also factors that are related to the respective condition (e.g. concomitant medications, symptoms) or therapy (e.g. side effects. complex treatment regimens) [7, 27]. In some conditions such factors may even be the most important cause of non-adherence. Moreover we analysed only selected factors. There are other therapy and condition unrelated factors that might have also an effect on adherence. E.g. for the physician- patient relationship, health literacy and positive beliefs about medication a positive effect on adherence is described for some chronic conditions. Again for long distance from treatment setting, forgetfulness and clinical asymptomatic course/disease mostly a negative influence on adherence is reported [3].

Most determinates of social status showed an effect on adherence. Apparently lower social status has a negative effect on adherence. This knowledge can contribute to identify vulnerable groups for non-adherence that are potential candidates for programs to enhance adherence [32]. The knowledge of adherence influencing factors can furthermore support the development of interventions and public health programs that are tailored to specific patient needs and that attempt to enhance adherence by reducing adherence barriers (e.g. counselling interventions tailored to education status, reduction of co-payments) [33].

Moreover the knowledge on adherence influencing factors can contribute to the development of risk factor based screening tools. Similar instruments have been developed for some indications [34].

## Conclusion

The presented overview indicates factors than can potentially have influence on adherence. Only for a few factors the influence on adherence was consistent. Belonging to an ethnic minority, unemployment and cost for the patient for their medications showed a negative effect on adherence which indicates that there is a social gradient. This indicates that future programs to enhance adherence should focus on social disadvantaged groups. For example a possibility could be to reduce co-payments for vulnerable groups.

Most of the other analysed factors showed heterogeneous results regarding statistical significance and/or effect direction. The results of this overview should therefore be considered as indication for factors that can have an influence on adherence in chronic physical conditions. To be of sufficient significance to make decisions in clinical practice, the factors have to be evaluated in detail for the specific context of the decision.

Further systematic reviews that assesses the risk of bias of included studies to allow an estimation of the validity of results and that are transparently reported are needed. Especially on the factors for that the evidence for effect were mostly rated unclear further research is needed.

## Electronic supplementary material

Additional file 1: Search strategy.(DOCX 16 KB)

Additional file 2: Results of individual systematic reviews.(DOCX 15 KB)

Below are the links to the authors’ original submitted files for images.

Authors’ original file for figure 1
