# Supplementary material for: Adherence influencing factors – a systematic review of systematic reviews
Source: Arch Public Health. 2014 Oct 27;72:37. doi: 10.1186/2049-3258-72-37 (PMC4323150; doi:10.1186/2049-3258-72-37)
Supplement: Supplementary file 1 — Additional file 1: Search strategy.(DOCX 16 KB) [file 13690_2014_5053_MOESM1_ESM.docx]

Supplement I: search strategy

| **Database (provider)** | **Search strategy** |
| --- | --- |
| **Embase (Embase)** | (adherence:ab,ti OR adherent:ab,ti OR adhere:ab,ti OR nonadherence:ab,ti OR nonadherent:ab,ti OR compliance:ab,ti OR 'patient compliance'/exp OR compliant:ab,ti OR comply:ab,ti OR noncompliance:ab,ti OR noncompliant:ab,ti) AND ('risk factor'/exp OR factor:ab,ti OR factors:ab,ti OR predict:ab,ti OR predictor:ab,ti OR predictors:ab,ti OR indicate:ab,ti OR indicator:ab,ti OR indicators:ab,ti OR influence:ab,ti OR influencing:ab,ti OR determinate:ab,ti OR determinates:ab,ti OR determination:ab,ti OR barrier:ab,ti OR barriers:ab,ti OR facilitate:ab,ti OR facilitator:ab,ti OR facilitators:ab,ti OR hindrance:ab,ti OR hindrances:ab,ti) AND ('drug therapy'/exp OR pharmacotherap*:ab,ti OR drug*:ab,ti OR pill*:ab,ti OR medicament*:ab,ti OR medication*:ab,ti OR medicin*:ab,ti OR pharmaceutical*:ab,ti OR tablet*:ab,ti OR treatment:ab,ti OR therapy:ab,ti) AND ('meta analysis'/exp OR 'systematic review'/exp OR (meta NEAR/1 analy*):ab,ti OR metaanalys*:ab,ti OR (systematic NEAR/1 (review* OR overview*)):ab,ti OR 'systematic literature review':ab,ti OR 'reference lists':ab OR bibliograph*:ab OR (hand NEXT/1 search*):ab OR (manual NEXT/1 search*):ab OR 'relevant journals':ab OR ('data extraction':ab OR 'selection criteria':ab OR 'inclusion criteria':ab AND review:ab,ti)) AND (english:la OR german:la) AND [embase]/lim AND [1990-2013]/py AND ('article'/it OR 'article in press'/it OR 'review'/it) |
| **MEDLINE (PubMed)** | (Adherence [TIAB] OR adherent [TIAB] OR adhere [TIAB] OR nonadherence [TIAB] OR nonadherent [TIAB] OR Compliance [TIAB] OR “patient compliance” [MeSH Terms] OR compliant [TIAB] OR comply [TIAB] OR noncompliance [TIAB] OR noncompliant [TIAB])  AND (risk factors [mesh] OR factor [TIAB] OR factors [TIAB] OR predict [TIAB] OR predictor [TIAB] OR predictors [TIAB] OR indicate [TIAB] OR indicator [TIAB] OR indicators [TIAB] OR influence [TIAB] OR influencing [TIAB] OR determinate [TIAB] OR determinates [TIAB] OR determination [TIAB] OR barrier [TIAB] OR barriers [TIAB] OR facilitate [TIAB] OR facilitator [TIAB] OR facilitators [TIAB])  AND (“drug therapy” [MeSH Terms] OR Pharmacotherap* [TIAB] OR Drug* [TIAB] OR pill* [TIAB] OR medicament* [TIAB] OR medication* [TIAB] OR medicin* [TIAB] OR pharmaceutical* [TIAB] OR tablet* [TIAB] OR treatment [TIAB] OR therapy [TIAB])  AND ("Meta-Analysis" [Publication Type] OR "Meta-Analysis as Topic" [Mesh] OR meta analy* [TIAB] OR metaanaly* [TIAB] OR systematic review* [TIAB] OR systematic literature review* [TIAB] OR systematic overview* [TIAB] OR "Review Literature as Topic" [Mesh] OR reference list* [TIAB] OR bibliograph* [TIAB] OR hand-search* [TIAB] OR relevant journals [TIAB] OR manual search* [TIAB] OR ((selection criteria [TIAB] OR inclusion criteria [TIAB] OR data extraction [TIAB]) AND review [Publication Type])) NOT ("Comment" [Publication Type] OR "Letter" [Publication Type] OR "Editorial" [Publication Type]) AND (english [la] OR german [la]) AND ("1990/01/01"[Date - Publication] : "3000"[Date - Publication]) |
