# Supplementary material for: Adherence influencing factors – a systematic review of systematic reviews
Source: Arch Public Health. 2014 Oct 27;72:37. doi: 10.1186/2049-3258-72-37 (PMC4323150; doi:10.1186/2049-3258-72-37)
Supplement: Supplementary file 2 — Additional file 2: Results of individual systematic reviews.(DOCX 15 KB) [file 13690_2014_5053_MOESM2_ESM.docx]

Supplement II:

| ***Factor***  ***Study*** | **Results (effect direction; comparisons showing effect direction/effect size; significant comparisons showing effect direction/95% CI; total number of comparisons/heterogeneity)** |
| --- | --- |
| **Broekmans 2008** | Age: ↑;NR ; 2; NR  Duration of disease: NR; NA; 0; 1  Education: NR; NA; 0; 2  Gender: Women > men; NR ; 1; 2  Different medications: ↓ ;1; 1; 1  Different medications: ↑; 1;1; 1 |
| **Daley 2012** | Age: ↑; 2; 2; 2  Comorbidity (mental): ↓; NR; 3; NR  Different medications: ↓; NR; 2; NR  Education: ↑; NR; 2; NR  Frequency of intake: ↑; NR; 1; NR  Income: ↑; 1; 0; 1  Marital status / social support: ↑; 1; 0; 1 |
| **Oosterom-Calo 2013** | Age: ↑; NR; 3; 7  Age: 35-56 > others; 1; 1; 1  Comorbidities (mental): ↓; 2; 2; 3  Comorbidities (mental): ↕; 1; 1; 3  Comorbidities (physical): ↑; NR; 3; 7  Comorbidities (physical): ↓; NR; 2; 7  Comorbidities (physical): ↕; 1; 1; 7  Different medications: NR; NA; 1; 2  Education: NR; NA; NR; 2  Ethnic status: ethnic minorities (e.g. African Americans) < majority ethnic groups (e.g. Caucasians); NR; 3; 5  Financial status: NR; NA; 0; 1  Frequency of intake: NR; NR; 0; 1  Gender: Men > women: NR; 3; 7  Gender: Women > men: NR; 2; 7  Number of pills taken per day: NR; NR; 1; 2  Social support: ↕; 2; 2; 2 |
| **Pasma 2013** | Age: ↑; NR; 3; 10  Age: 55-64 > others; 1; 1; 10  Comorbidity: ↑; NR; 2; 5  Co-payments: ↓; 1; 1; 1  Different medications: ↑; NR; 1; 3  Duration of disease: ↓; NR; 1; 9  Employed fulltime: NR; 3; 3; 3  Ethnic status: white > others; 1; 1; 1  Frequency of intake: NR; NR; 0; 2  Gender: Men > Women: NR; 1; 10  Gender: Women > men: NR; 1; 10  Medication costs: ↓; 1; 1; 1  Single / divorced: ↓; NR; 1; 4  Social support: ↑; NR; 1; 4  Socioeconomic status: ↓; NR; 1; 4 |
| **Schrijvers 2013** | Age: ↓; NR; 1; 2 |
| **Sinott 2013** | No co-payments vs. co-payments; OR = 1.11; 1.09 to 1.14; p=0.37, I^2^ = 7% |
| **Verbrugghe 2012** | Age: <45 < others; 1; 1; NR  Age: ≤45 or ≥85 vs. others; 1; 1; NR  Age: ↑; 1; 1; NR  Age: ↓; 2; 2; 2  Comorbidity: ↓; 2; 2; NR  Co-payments: ↓; 2; 2; NR  Different medications: ↓; 2; 2; NR  Duration of disease: ↓; 1; 1; NR  Duration of therapy: ↓; 3; 3; NR  Education: ↑; 1; 1; NR  Ethnic status: African American > others; 1; 1; NR  Ethnic status: non-white > others; 1; 1; NR  Gender: men > women; 1; 1; NR  Gender: women > men; 1; 1; NR  Income: ↑; 1; 1; NR  Living alone < others; 1; 1; NR  Marital status: non-married > married; 1; 1; NR  Medication costs: ↓; 1; 1; NR  Taking medication not at meal times; ↓; 1; 1; NR |
