# Supplementary material for: Adherence influencing factors – a systematic review of systematic reviews
Source: Arch Public Health. 2014 Oct 27;72:37. doi: 10.1186/2049-3258-72-37 (PMC4323150; doi:10.1186/2049-3258-72-37)
Supplement: Supplementary file 3 — Authors’ original file for figure 1 [file 13690_2014_5053_MOESM3_ESM.pdf]

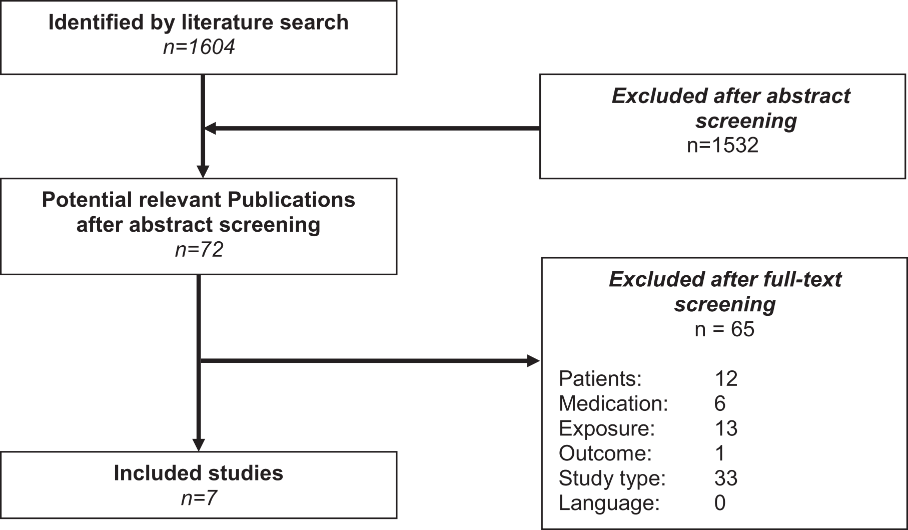
Identified by literature search
n=1604
Excluded after abstract screening
n=1532
Potential relevant Publications after abstract screening
n=72
Excluded after full-text screening
n = 65
Patients: 12
Medication: 6
Exposure: 13
Outcome: 1
Study type: 33
Language: 0
Included studies
n=7
